# Molecular Mechanisms of Disease Pathogenesis Differ in Krabbe Disease Variants

**DOI:** 10.1111/tra.12404

**Published:** 2016-05-30

**Authors:** Samantha J. Spratley, Chris H. Hill, Agnete H. Viuff, James R. Edgar, Karsten Skjødt, Janet E. Deane

**Affiliations:** ^1^ Cambridge Institute for Medical Research, Department of PathologyUniversity of CambridgeCambridgeCB2 0XYUK; ^2^ Current address: MRC Laboratory of Molecular BiologyCambridgeCB2 0QHUK; ^3^ Department of ChemistryAarhus UniversityAarhus C8000Denmark; ^4^ Cambridge Institute for Medical Research, Department of Clinical BiochemistryUniversity of CambridgeCambridgeCB2 0XYUK; ^5^ Department of Cancer and InflammationUniversity of Southern DenmarkOdense5000Denmark

**Keywords:** galactocerebrosidase, globoid cell leukodystrophy, glycosphingolipid, Krabbe disease, lysosomal storage disease

## Abstract

Krabbe disease is a severe, fatal neurodegenerative disorder caused by defects in the lysosomal enzyme galactocerebrosidase (GALC). The correct targeting of GALC to the lysosome is essential for the degradation of glycosphingolipids including the primary lipid component of myelin. Over 100 different mutations have been identified in GALC that cause Krabbe disease but the mechanisms by which they cause disease remain unclear. We have generated monoclonal antibodies against full‐length human GALC and used these to monitor the trafficking and processing of GALC variants in cell‐based assays and by immunofluorescence microscopy. Striking differences in the secretion, processing and endosomal targeting of GALC variants allows the classification of these into distinct categories. A subset of GALC variants are not secreted by cells, not proteolytically processed, and remain trapped in the ER; these are likely to cause disease due to protein misfolding and should be targeted for pharmacological chaperone therapies. Other GALC variants can be correctly secreted by cells and cause disease due to catalytic defects in the enzyme active site, inappropriate post‐translational modification or a potential inability to bind essential cofactors. The classification of disease pathogenesis presented here provides a molecular framework for appropriate targeting of future Krabbe disease therapies.

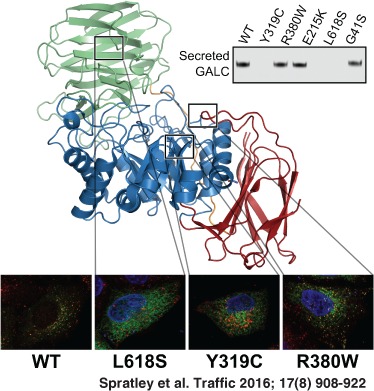

Krabbe disease, also known as globoid cell leukodystrophy, is a rare, autosomal recessive disorder in which extensive demyelination is accompanied by rapid, fatal neurodegeneration. Krabbe disease is caused by defects in the lysosomal enzyme galactocerebrosidase (GALC) that is essential for the catabolism of galactosphingolipids, including the primary lipid component of myelin, galactocerebroside. The molecular understanding of Krabbe disease has been aided by the sequence characterization of the human gene, structure determination of the GALC protein and via the study of various animal models of the disease 1, 2, 3, 4, 5. The GALC protein is produced and glycosylated in the endoplasmic reticulum (ER) and trans‐Golgi network (TGN) where it is post‐translationally modified by N‐linked glycosylation at four sites 3. GALC then becomes cargo for the cation‐independent mannose‐6‐phosphate receptor (M6PR) which targets it to lysosomes either directly from the trans‐Golgi network (TGN) or indirectly via secretion and re‐uptake 6. It is in the endolysosome/lysosome that GALC processes lipid substrates. Sphingolipid degradation by lysosomal hydrolases also requires non‐enzymatic sphingolipid activator proteins known as saposins. In rare cases, Krabbe disease can be caused by lack of functional saposin A 7.

Despite these molecular insights into GALC function the cellular events that result in apoptosis of myelin‐forming oligodendrocytes and Schwann cells of the central and peripheral nervous systems remains poorly understood. Krabbe disease severity and progression are highly variable and the age of clinical onset can vary from the first few weeks of life (early infantile) through late‐infantile and juvenile into adult presentations (late‐onset). To date, 147 different mutations in the *GALC* gene have been catalogued for the Online Metabolism and Molecular Bases of Inherited Disease (OMMBID) 8.

Currently, the only available treatment for Krabbe disease is haematopoietic stem cell transplantation but as this cannot repair damage that has already occurred it must be initiated before the onset of symptoms. For this reason early diagnosis is crucial and has led to the establishment of several newborn screening programmes 9. For mutations of known severity these screening programmes aid the rapid implementation of treatment. However, for newly identified mutations the clinical severity remains uncertain.

Understanding the pathogenesis of specific mutations in GALC is not only important for predicting the severity of newly identified mutations but is also important for determining which patients will be appropriate candidates for new treatments and combination therapies currently being developed 10, 11, 12, 13. One such therapy being pursued for Krabbe disease and a number of related lysosomal storage diseases is pharmacological chaperone therapy 14, 15, 16, 17, 18. The goal of this approach is to treat patients with small molecules that can bind and stabilize partially misfolded enzyme that would otherwise remain trapped in the ER and be degraded by cellular quality‐control pathways. This approach will not be appropriate for patients with large deletions in the *GALC* gene or for variants that are catalytically defective but must instead be targeted towards missense mutations that cause protein misfolding. For these reasons, it is important that we understand the molecular defects caused by specific mutations in GALC. Here we present the characterization of a series of Krabbe disease variants and highlight different molecular mechanisms underlying the pathogenesis of this disease.

## Results

### Missense mutations alter GALC secretion and processing

**Figure 1:**
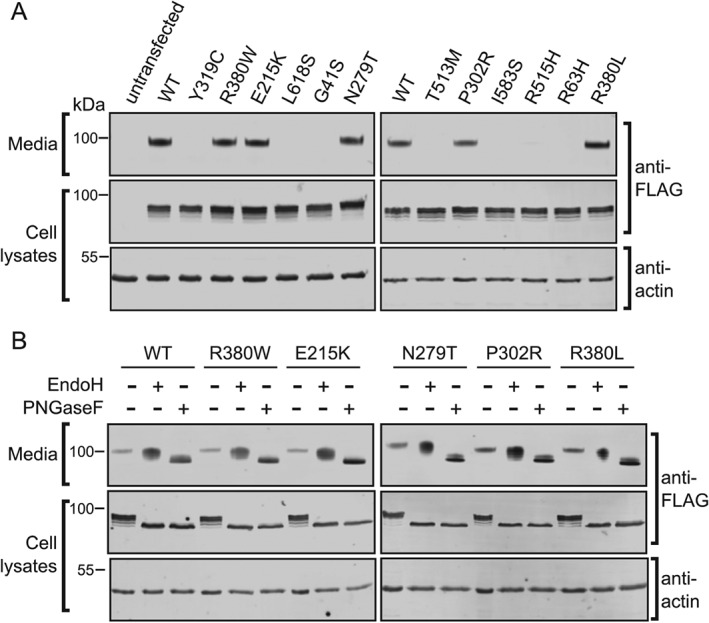
**Missense mutations alter GALC secretion from cells**. HEK293T cells were transiently transfected with FLAG‐tagged wild‐type (WT) GALC and a panel of clinically relevant Krabbe disease mutations. Conditioned media was harvested at 72 h and cells lysed in 1% SDS followed by SDS‐PAGE and immunoblotting. A) Secreted and total GALC expression were detected using western blot with an anti‐FLAG antibody. Actin served as a loading control. B) Conditioned media and cell lysates were digested with either EndoH or with PNGaseF before immunoblotting with anti‐FLAG‐to detect GALC.

In order to understand the different effects of missense mutations on GALC processing and trafficking a series of clinically relevant variants were selected and expressed in HEK293T cells. Two numbering schemes are commonly used for GALC based on either the first or second methionine in the ER signal sequence. Here we are using the numbering based on the second methionine as this is consistent with the majority of the literature and the available structural data. For clarity we have included a table showing the equivalent numbering for the alternative start site (Table S1, Supporting Information). FLAG‐tagged GALC variants were detected in cell lysates and conditioned media by western blot. Use of the anti‐FLAG antibody in this case ensures that all GALC variants are detected using an identical epitope reducing any differences in binding of the antibody due specifically to the encoded mutation. All GALC variants are equally expressed in cells and individual bands are detected in cell lysates representing the addition of four N‐linked glycans as GALC is produced and glycosylated in the ER and TGN (Figures 1A and 5B). However, only a subset of these variants is secreted and detected in the conditioned media suggesting a defect in trafficking beyond the ER‐TGN (Figure 1A). For those variants that are secreted, only one band is detectable in the conditioned media identifying that these proteins possess uniform glycosylation and are therefore likely to have undergone correct processing before being secreted from cells. To confirm this, the sensitivity of GALC glycans to PNGaseF and EndoH was tested (Figure 1B). The partially processed protein in the cell lysates was susceptible to both treatments as expected, while the protein secreted into the media was primarily EndoH resistant confirming that the glycans had been correctly modified during their trafficking through the ER‐TGN.

**Figure 2:**
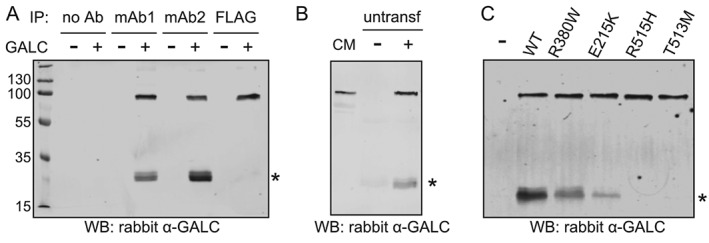
**Proteolytic cleavage of WT and mutant GALC**. A) Protein lysates from either untransfected HEK293T cells or from cells transfected with WT GALC were subjected to immunoprecipitation with monoclonal antibodies against full‐length human GALC (mAb1 and mAb2) or the FLAG epitope tag. GALC was detected using western blot with a polyclonal rabbit GALC antibody, cleavage product at ∼30‐kDa is marked with an asterisk. B) Conditioned media (CM) from HEK293T cells transfected with WT GALC contains full‐length GALC (lane 1). GALC‐containing CM (+) and CM from untransfected cells (−) was harvested after 72 h and applied to untransfected HEK293T cells for 48 h. Treated cells were subjected to immunoprecipitation using the GALC monoclonal antibody mAb2 and detected as in A. C) HEK293T cells were transiently transfected with WT GALC and Krabbe mutations R380W, E215K, R515H and T513M and immunoprecipitated using the mAb2 antibody and detected as in A.

The secretion profile illustrated here for a range of missense mutations provides an initial classification of GALC variants into those that are correctly processed and secreted and those that may cause disease due to misfolded protein being trapped in the ER. To confirm the delivery, or lack thereof, to the lysosomal compartment there is an additional post‐translational processing step that occurs in the lysosome. Upon delivery to the lysosome, a loop on the surface of GALC is cleaved by a lysosomal protease. This event does not affect activity and is not likely to have any effect on the structure but can be detected by SDS‐PAGE as 50‐ and 30‐kDa bands 6, 19, 20. In the previous assays (Figure 1A,B) there was no detectable band for the lower molecular weight cleavage product expected upon delivery of GALC to the lysosome. However, as detection of GALC involved using an antibody against the epitope tag it was possible that in the proteolytic environment of the endolysosomal system this tag is cleaved from the GALC protein restricting detection of the processed form. To overcome this, we generated a new panel of monoclonal antibodies raised against the full‐length human GALC protein. We carried out an immunoprecipitation (IP) of GALC from cell lysates using either the FLAG antibody or two different mouse monoclonal antibodies (mAb1, mAb2). Following IP, bands were detected by western blot using a rabbit polyclonal antibody raised against a peptide within the smaller C‐terminal 30‐kDa fragment (Figure 2A). For all samples the full‐length uncleaved GALC is detectable at approximately 80 kDa. In both the IPs carried out with monoclonal antibodies an additional band is present at 30‐kDa consistent with the fragment size following cleavage upon delivery to the lysosome. This band is not detectable in the IP using the anti‐FLAG antibody highlighting the critical importance of appropriate antibodies for assay development. To confirm that the cleavage event occurs post‐Golgi and thus is a measure of successful trafficking to lysosomes we carried out an uptake assay whereby conditioned media containing full‐length uncleaved GALC was applied to untransfected HEK293T cells (Figure 2B). The appearance of the 30‐kDa fragment in untransfected cells treated with full‐length GALC identifies that the detection of cleaved GALC can provide a measure of lysosomal delivery of wild‐type (WT) and mutant forms of GALC. Cell lysates from GALC missense mutations that are not secreted into the conditioned media, such as R515H and T513M (Figure 1A), have no detectable 30‐kDa cleavage product (Figure 2C) consistent with these proteins being trapped in the ER due to misfolding and therefore are not delivered to lysosomes. Missense mutations that are secreted into the conditioned media, such as R380W and E215K, show reduced but still detectable levels of GALC cleavage product.

### Non‐secreting, unprocessed missense mutations of GALC are trapped in the ER

**Figure 3:**
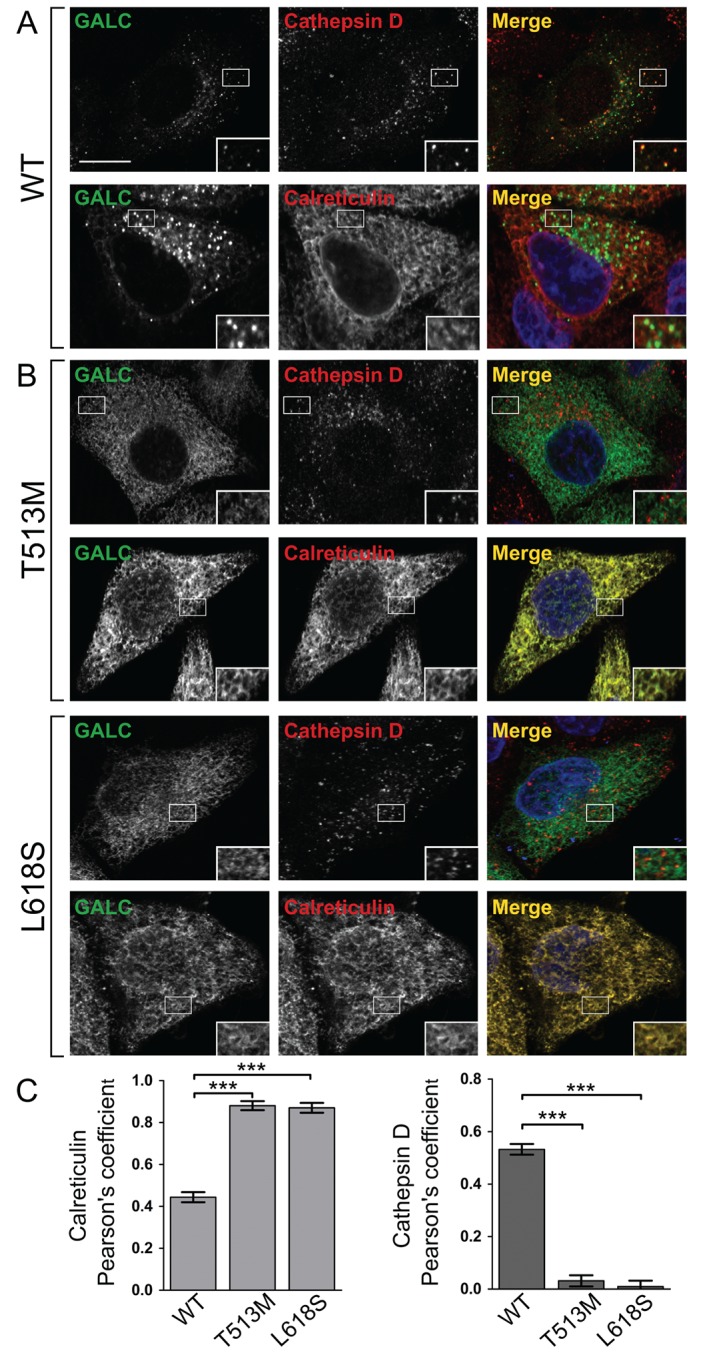
**Missense mutations of GALC that interfere with secretion and cleavage are trapped in the ER**. Representative confocal microscopy images of HeLa cells transiently transfected with either WT GALC (A), or the Krabbe mutations T513M and L618S (B). Cells were plated onto glass coverslips, fixed and immunostained using monoclonal antibody against GALC (green), the lysosomal marker cathepsin D (red) or the ER marker calreticulin (red). Nuclei were stained with DNA‐binding dye, DAPI (blue). Scale bar: 10 µm. C) To quantify colocalization, Pearson's correlation coefficients were calculated for each variant with both cathepsin D and calreticulin. Mean ± SEM for at least 20 individual cells from ≥3 independent experiments are shown. ***p ≤ 0.0001, calculated using a two‐tailed unpaired t‐test.

Absence of GALC in the conditioned media and the lack of cleaved product in cell lysates together provide compelling evidence that a particular GALC variant causes disease due to compromised protein folding and is thus retained in the ER. However, both these measures are indirect and so several GALC variants were further tested by direct monitoring of their cellular localization. GALC variants were expressed in HeLa cells and detected by immunofluorescence microscopy using monoclonal antibodies raised against full‐length human GALC and compared with markers for the ER (calreticulin, calnexin and REEP5) and lysosome (cathepsin D). Immunofluorescence microscopy clearly identifies that WT GALC co‐localizes with cathepsin D identifying its correct trafficking to lysosomes (Figure 3A). Missense mutations that were shown to inhibit secretion of GALC and prevent normal processing in the lysosome were tested for their cellular localization (Figure 3B, Figures S1 and S2). T513M and L618S show clear reticular staining consistent with ER architecture and very poor co‐localization with the lysosomal marker (Figure 3C). The co‐localization of these variants with the ER markers confirmed their ER localization (Figure 3B,C, Figure S1). Additional missense mutations I583S, R515H and Y319C were also identified to co‐localize with the ER markers calreticulin and calnexin (Figure S2).

**Figure 4:**
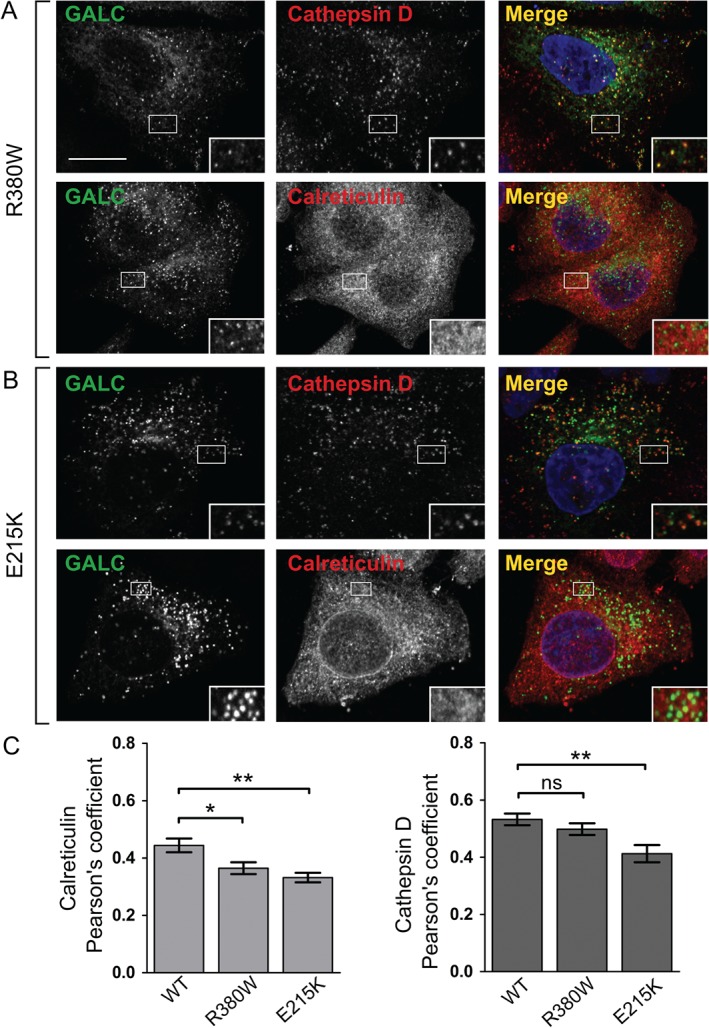
**Missense mutations R380W and E215K are trafficked to the lysosome**. Representative confocal microscopy images of HeLa cells transiently transfected with Krabbe mutations R380W (A) and E215K (B). Transfected cells were plated onto glass coverslips, fixed and immunostained for GALC (green), the lysosomal marker cathepsin D (red) or the ER marker calreticulin (red). Scale bar: 10 µm. C) Pearson's correlation coefficients were calculated for each of the mutations with both cathepsin D and calreticulin. Mean ± SEM for at least 20 individual cells from ≥3 independent experiments are shown. **p ≤ 0.005, *p ≤ 0.05, ns = not significant, calculated using a two‐tailed unpaired t‐test.

Missense mutations of GALC that are secreted by cells and show cleavage consistent with lysosomal localization were also tested for their cellular localization. The residue R380 has been shown by us previously to be critical for enzyme activity as it directly binds substrate in the active site 3, 21. In this case, we would expect this mutation not to interfere with folding and to traffic to the lysosome where the disease‐causing defect is primarily catalytic. In agreement with this, immunostaining of the R380W mutant identifies that it co‐localizes with lysosomes to the same extent as WT GALC (Figure 4A,C). GALC containing the missense mutation E215K can be secreted into the media and shows cellular localization similar to WT GALC (Figure 4B). Consistent with the secretion profile, E215K is not primarily co‐localized with the ER marker calreticulin suggesting that the disease mechanism in this case is not due to a misfolding defect (Figure 4C).

### N279T introduces a new glycosylation site that interferes with protein trafficking

**Figure 5:**
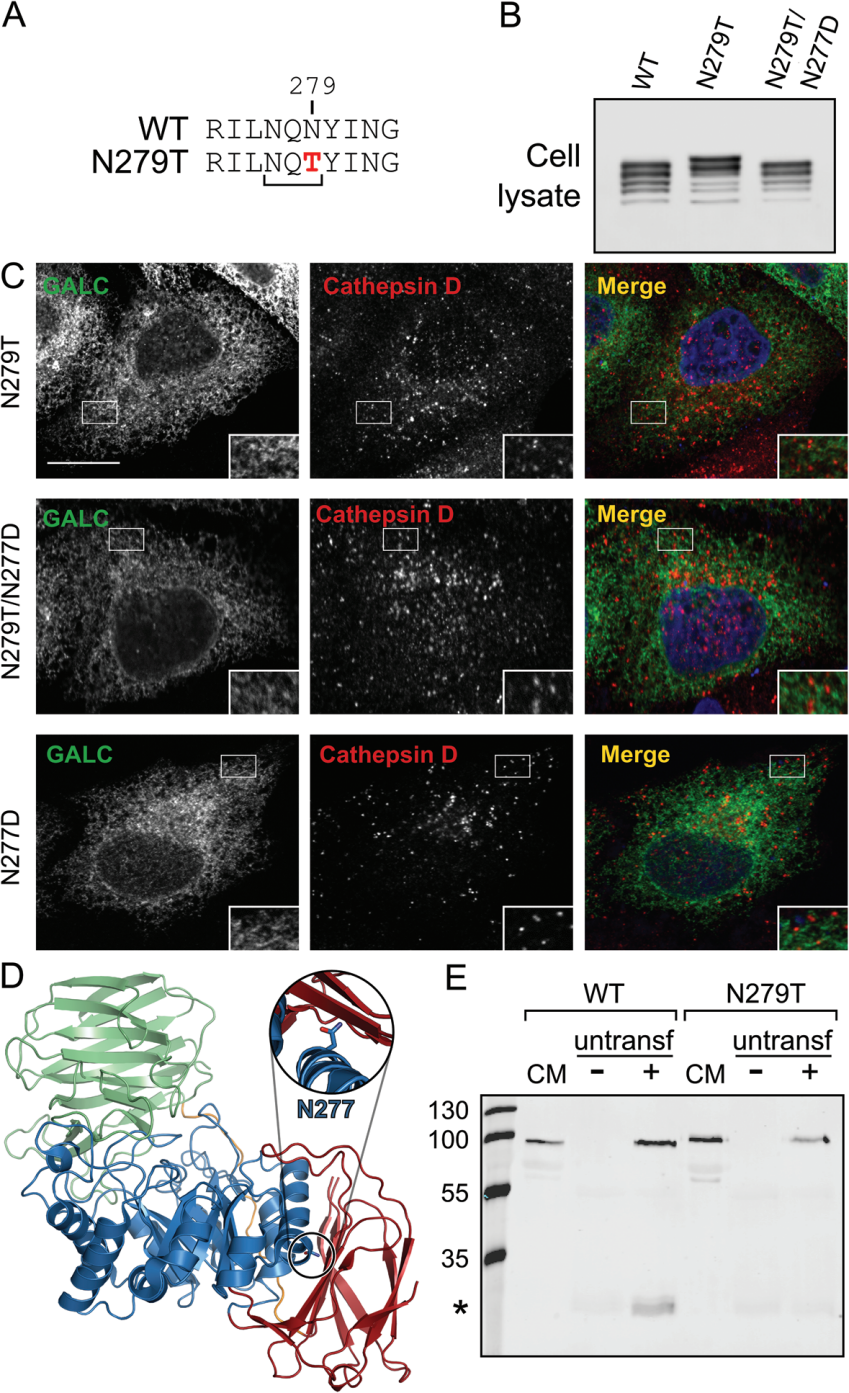
**N279T is a hyperglycosylation mutant with defective trafficking**. A) Protein sequence of WT and N279T GALC illustrating the introduction of a new consensus glycosylation site (NXT). B) HEK293T cells transiently transfected with WT GALC, Krabbe mutation N279T or double mutant N279T/N277D were lysed with 1% SDS, separated by SDS‐PAGE and immunoblotted. Total GALC expression was analysed using western blot against the FLAG epitope tag. C) HeLa cells were transiently transfected with Krabbe mutation N279T, the double mutation N279T/N277D or N277D alone, plated and fixed on glass coverslips and immunostained for GALC (green) and the lysosomal marker cathepsin D (red). Scale bar: 10 µm. D) The newly glycosylated residue, N277, is highlighted on the structure of GALC (PDB ID: 3ZR5). The structure is coloured according to domain (TIM barrel in blue, β‐sandwich in red and lectin domain in green). Zoomed view (inset) shows the N277 sidechain as sticks. E) Conditioned media from either untransfected HEK293T cells (−) or cells transfected with WT or Krabbe mutation N279T (+) was harvested after 72 h and applied to untransfected HEK293T cells for 48 h. Treated cells were subjected to immunoprecipitation using the GALC monoclonal antibody mAb2. GALC was detected using western blot with a polyclonal rabbit GALC antibody.

The missense mutation N279T is detected in conditioned media but runs at a slightly higher molecular weight compared with WT and other missense variants (Figure 1A). Analysis of the sequence surrounding this residue identifies that this mutation introduces a new consensus site for N‐linked glycosylation of residue N277 (Figure 5A). This additional glycan can be detected in cell lysates and can be abolished by introduction of the second mutation N277D to block glycosylation of this residue (Figure 5B). Unlike other secreted GALC variants, N279T does not localize to lysosomes (Figure 5C). Introduction of an additional glycan at residue N277 would be predicted to destabilize the fold of this GALC variant as this residue is deeply buried at the interface between two domains (Figure 5D). Although introduction of the second mutation N277D blocks addition of the extra glycan, the double mutant does not rescue trafficking to the lysosome (Figure 5C). Despite the conservative nature of the introduced N277D mutation, this change alone causes misfolding of GALC such that it is not trafficked to the lysosome (Figure 5C). The observation that N279T can be secreted but not enter the endocytic pathway suggests that some protein can escape ER‐associated quality control but is defective for binding to the M6PR. To test this, an uptake assay was carried out whereby conditioned media containing the N279T variant was applied to untransfected cells in a manner similar to that shown for WT GALC (Figure 5E). Unlike WT GALC, the N279T variant cannot be taken up by cells suggesting that the additional glycan interferes with endocytosis.

### The polymorphism at position 546 has subtle effects on enzyme activity, stability and trafficking

**Figure 6:**
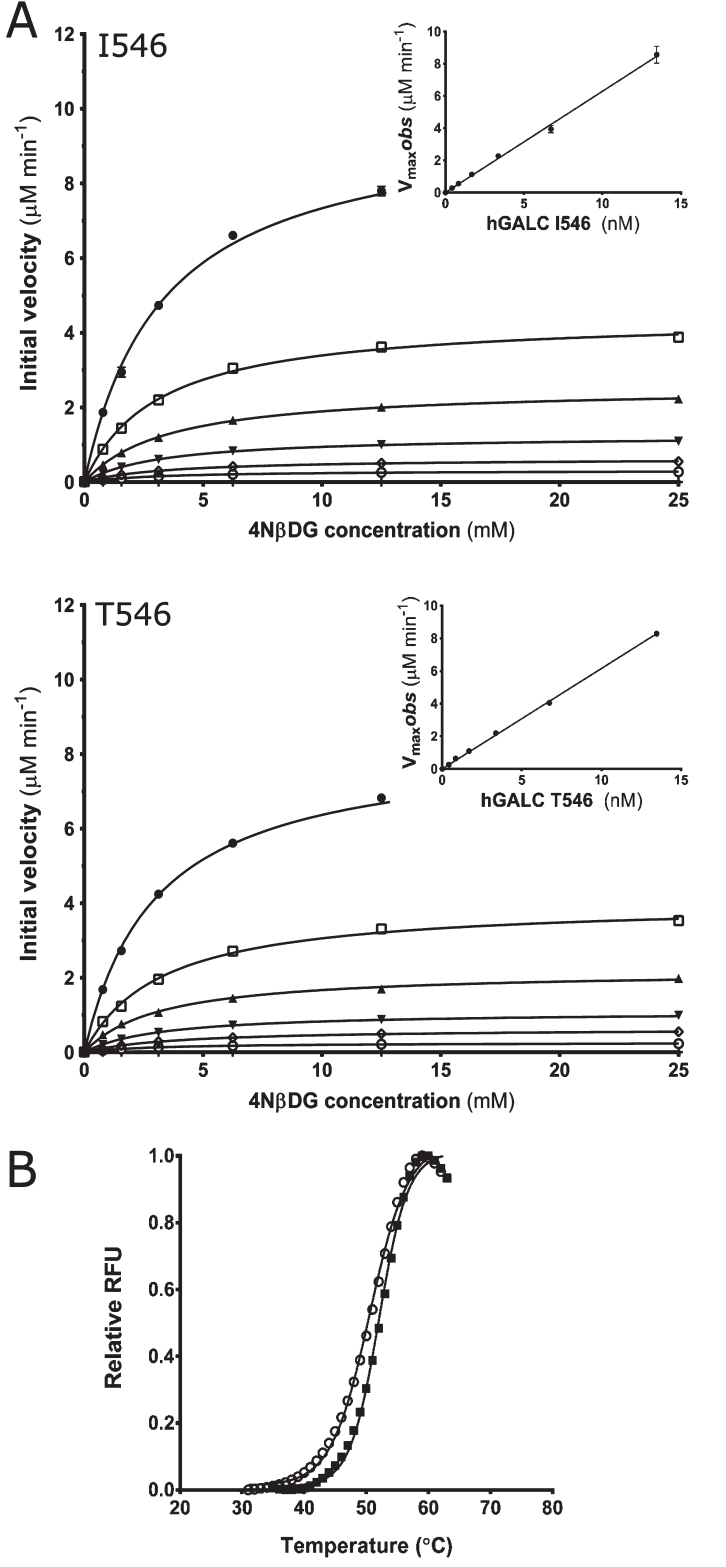
**Effect of the I546T polymorphism on enzyme activity and stability**. A) Michaelis–Menten plots of initial velocity versus substrate concentration at GALC concentrations of 0.420 nm (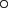), 0.842 nm, (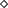), 1.68 nm, (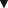), 3.37 nm, (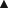), 6.73 nm, (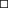), and 13.4 nm (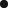). (Inset) Plot of V_max_ observed versus GALC concentration showing k_cat_ as the gradient (11.9/sec for I546 and 10.6/sec for T546). Experiments were performed in triplicate and SEM error bars are shown. B) Differential scanning fluorimetry of GALC polymorphisms I546 (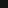) and T546 (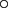) demonstrate similar thermal denaturation profiles.

**Figure 7:**
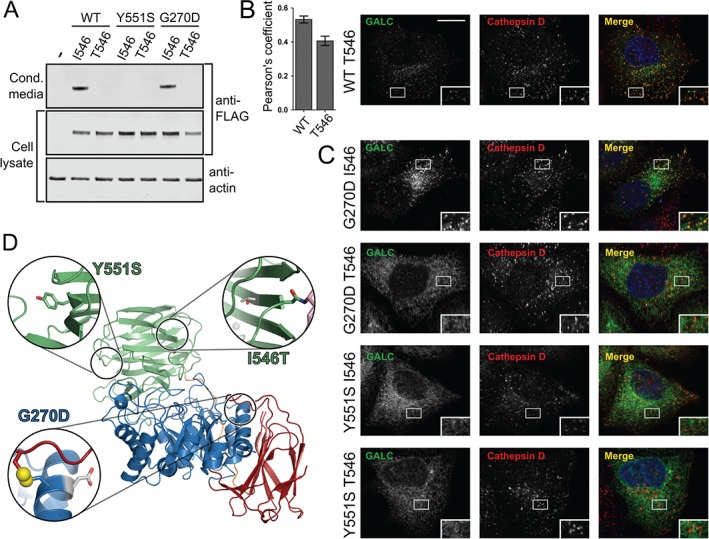
**Polymorphic background alters the trafficking of some missense mutations**. A) HEK293T cells were transiently transfected with WT GALC (I546), T546, Y551S‐I546, Y551S‐T546, G270D‐I546, G270D‐T546. Conditioned media was harvested at 72 h and cells lysed in 1% SDS followed by SDS‐PAGE and immunoblotting. Secreted and total GALC expression was detected using western blot against the FLAG epitope tag and loading analysed by blotting against actin. B) Representative confocal microscopy images of HeLa cells transiently transfected with WT GALC containing the T546 polymorphism. Cells were plated onto glass coverslips, fixed and immunostained for GALC (green), the lysosomal marker cathepsin D (red). Nuclei were stained with DNA‐binding dye, DAPI (blue). Scale bar 10 µm. To quantify colocalization Pearson's correlation coefficients were calculated for cathepsin D. Mean ± SEM for at least 20 individual cells from ≥3 independent experiments are shown. C) Equivalent confocal microscopy images as in panel B for Y551S‐I546, Y551S‐T546, G270D‐I546 and G270D‐T546. D) The positions of I546T, G270D and Y551S are highlighted on the structure of GALC (PDB ID: 3ZR5). The structure is coloured as in Figure 5. For each variant, the zoomed view (inset) shows the relevant side chain as sticks and the surrounding region of the structure that would be affected by the mutation. The single disulphide bond (yellow) present in GALC is illustrated as spheres.

The missense mutations described here have been tested in the most common polymorphic background I546. However the polymorphism T546 has been reported to be present within the population at allele frequency of 40–45% 22. Clinical assays for GALC activity in peripheral blood have suggested that the T546 variant has half the activity of the I546 variant, contributing to the highly variable ‘normal’ activity levels within the general population 22, 23, 24. In order to test the effect of this polymorphism on GALC activity we generated stable expression constructs of each of these polymorphisms and selected high‐yield clones in order to purify milligram quantities of GALC for steady‐state kinetic studies (Figure 6A and Table S2). Surprisingly, the two variants do not have significantly different catalytic properties *in vitro*: T546 has 89% activity of I546 suggesting that the observed differences in activity from blood samples may be due to differential stability, expression or trafficking in cells. Thermal denaturation of secreted GALC monitored by differential scanning fluorimetry DSF revealed a subtle destabilization of the T546 variant *in vitro* (Figure 6B; *T*_*m*,_ I546 = 51.8 ± 0.2°C versus *T*_*m*,_ T546 = 50.6 ± 0.2°C). To determine whether this degree of destabilization was sufficient to impair trafficking of GALC the presence of T546 in conditioned media was monitored. Interestingly, this variant was not detectable in the media suggesting it is trapped in the ER (Figure 7A). However, if this polymorphism completely blocked trafficking of GALC to the lysosome this would represent a disease‐causing mutation. Cellular localization of T546 by immunofluorescence microscopy confirms that although this variant is not detected in conditioned media it can be trafficked to the lysosome (Figure 7B). The combination of multiple, subtle deleterious effects on activity, stabilization and trafficking of the T546 variant may explain the observed reduction in enzyme activity from peripheral blood samples.

Although the I546T polymorphism on its own has only subtle effects on GALC function, when combined with additional missense mutations the cumulative effects can become severe. Indeed, it has been suggested previously that certain mutations are only disease‐causing in specific polymorphic backgrounds 25. The G270D variant is responsible for a late‐onset form of Krabbe disease and is usually found in *cis* with the I546T polymorphism 26. In the I546 background, the G270D variant possesses WT levels of protein secreted into the media and WT localization to lysosomes (Figure 7A,C). However, in the T546 background, the G270D mutation is severely compromised in its cellular trafficking and is not localized to lysosomes (Figure 7A,C, Figure S3). The cumulative effect of these two sequence variations is not due to a single localized structural perturbation as the two residues, G270 and I546, lie in distant parts of the GALC structure (Figure 7D). Specifically, G270 is situated near the disulphide bond that stabilizes a critical active‐site loop structure while residue 546 lies in the C‐terminal lectin domain.

The missense mutation Y551S causes infantile Krabbe disease and has been identified in the T546 polymorphic background 26. These two residues are much closer to each other in the GALC structure and so might be expected to have a severe cumulative effect on folding (Figure 7D). However, the secretion profile of this mutation shows that even in the I546 background the Y551S mutation has a severe detrimental effect on protein folding and secretion (Figure 7A). In agreement with this, the Y551S variant in both polymorphic backgrounds does not traffic correctly to lysosomes (Figure 7C and Figure S3).

## Discussion

In order to better understand the underlying molecular mechanisms causing GALC dysfunction in Krabbe disease, clinically relevant variants of GALC were expressed in cells and monitored for their capacity to traffic beyond the ER‐TGN and to localize to their site of action in lysosomes. Using a series of cell‐based assays we have identified the underlying pathogenesis for a number of these variants. Initial screening of the secretion profile of GALC variants provides a good first indication of whether a mutation may result in protein misfolding. The protein quality control pathways present in the ER should result in the retention and degradation of misfolded protein. In this case misfolded GALC will not traffic beyond the ER and thus will not be detected in the conditioned media. Using this approach we identified seven variants (Y319C, L618S, G41S, T513M, I583S, R515H and R63H) that are likely to cause disease due to protein misfolding. Another subset of GALC variants (R380W, E215K, N279T, P302R and R380L) were present in the conditioned media identifying that the underlying pathology in these cases is not primarily protein misfolding. A further assay was used to monitor trafficking beyond the ER‐TGN by exploiting the observation that upon delivery of GALC to lysosomes, a 30‐kDa cleavage product can be identified following SDS‐PAGE and immunoblotting. In support of their predicted defect in protein folding, two of the missense mutations identified as non‐secretors, R515H and T513M, were not processed into this smaller fragment. Consistent with the secretion assay, two of the secreted variants (R380W and E215K) were shown to undergo proteolytic processing similar to that seen for WT GALC.

Lack of protein secretion and the inability to be processed into the 50‐ and 30‐kDa fragments are indicative of a misfolded GALC variant suggesting it is retained in the ER. To further test this, the cellular localization of GALC variants was directly monitored by immunofluorescence microscopy using newly generated, specific monoclonal antibodies raised against full‐length human GALC protein. Using this approach, the GALC variants, T513M, L618S, Y319C, I583S and R515H were not localized to lysosomes and were trapped in the ER (Figure 3, Figure S1 and S2). These GALC variants can be classified as protein misfolding mutants due to their lack of secretion, inability to be proteolytically processed and their retention in the ER.

Those disease variants that do not result in significant misfolding, based on their secretion, processing and cellular localization, fall into a number of categories. The residue R380 has been shown by us previously to play a critical role in the catalytic processing of substrates 3, 21. In support of this, R380W and R380L are secreted similarly to WT GALC and R380W was shown to localize to lysosomes. E215K was also normally secreted and trafficked within the cell but is not likely to be catalytically defective as this mutation does not lie near the enzyme active site. This residue is on the surface of GALC and the charge inversion caused by this mutation may interfere with important interactions with partner proteins such as saposin A. The mutation N279T was shown to introduce a new glycosylation consensus sequence resulting in the addition of an N‐linked glycan to residue N277. This variant is capable of being secreted by cells and so can pass ER quality control but is unable to be taken up by cells or traffic to lysosomes suggesting that the new glycan may interfere with endocytosis by altering binding to the M6PR.

A number of polymorphisms have been identified in the *GALC* gene and have been shown to have a negative effect on GALC activity. In this study we produced both the I546 and T546 variants of GALC and compared their activity and stability *in vitro*. Surprisingly this polymorphism has only a subtle effect on GALC enzyme kinetics and thermal stability but did have a significant effect on its trafficking beyond the ER‐TGN. This suggests that the lower activity reported for this polymorphism is a reflection of its reduced trafficking rather than its inability to process substrate. It has been shown previously that the polymorphic background that a missense mutation occurs can critically change the disease pathogenesis 25, 27. In agreement with this, we show that G270D only results in protein misfolding, as measured by ER co‐localization, when present in the T546 background. Thus in this case, it is the combination of two subtle defects that results in disease. However, despite the Y551S variant only being identified in the T546 background, it results in protein misfolding in both polymorphic backgrounds identifying that this mutation alone is sufficient to cause significant protein misfolding.

The availability of structural data for GALC allows prediction of the functional effects of missense mutations: the greater proportion of a side chain that is buried in the structure the greater the likelihood that a mutation of that residue will disrupt the fold 3. These predictions can now be compared with the functional effects identified here (Table S3). The side chains for residues G41, Y319, T513, I583 and L618 are completely buried in the structure suggesting that mutation of these would result in misfolding and in agreement with this prediction, all were not secreted by cells and/or were localized to the ER. However, G270 is also completely buried but the G270D variant is only localized to the ER when present in the T546 background. The side chains of R63, R515 and Y551 are also primarily (∼80%) buried in the structure and are here identified as non‐secretors identifying that even only partially buried residues can significantly disrupt folding. However, based on buried surface area, P302 would also fall into this category but is shown here to be secreted in disagreement with the structure‐based prediction. E215 and R380 are relatively exposed on the surface of GALC (only 57% and 48% buried) suggesting that residues that are <60% buried are unlikely to result in misfolding. On the basis of this analysis, the structure provides a good molecular framework for making predictions but verification in cell‐based assays is still required.

In this study we have used three different assays to monitor trafficking defects in GALC variants. Each assay has different levels of complexity and sensitivity allowing a confidence level to be associated with the designation of the molecular defect caused by a specific mutation. The importance of using multiple assays is best highlighted by those variants that have subtle effects such as the I546T polymorphism. Although this polymorphism is not disease‐causing, in our secretion assay it is not detectable suggesting that this assay alone is not sufficiently sensitive to distinguish subtle GALC defects. However, monitoring cellular localization by immunostaining appears to be much more sensitive as in this case the polymorphic variant can be localized to lysosomes. Thus although the secretion assay is a fast and convenient readout it is important that conclusions regarding the underlying pathogenesis of subtle variants be confirmed using the more sensitive co‐localization assay.

Extrapolation from disease mechanism to disease severity remains highly challenging. The disease‐causing variant L618S has a complex effect in patients and has been identified in patients with age of onset ranging from late‐infantile to adult 28, 29, 30. This variation in disease severity is likely influenced by many factors including the polymorphic background and compound heterozygosity both of which have been documented for this variant. Recently, Shin et al. also showed that L618S is severely compromised in its ability to be correctly processed into the 50‐ and 30‐kDa fragments supporting a defect in folding 31. However, unlike our localization studies identifying L618S as trapped in the ER, Shin et al. found some evidence that this variant co‐stains with LAMP2 suggesting it maintains some capacity to be delivered to late endosomes/lysosomes. For this reason one must be cautious to assign degree of disease severity based on these assays but should instead use the identification of the nature of the defect as a critical factor for subsequent therapeutic targeting. Mutations that cause misfolding, giving rise to lack of secretion, processing and primarily ER co‐localization, may be good targets for pharmacological chaperone therapies while those that retain significant capacity for correct trafficking and processing will require future enzyme replacement approaches. However, the extent of misfolding caused by specific mutations will alter the effectiveness of pharmacological chaperones. Specifically, the misfolding caused by some mutations may prove too severe to respond to these approaches and will therefore require enzyme replacement strategies.

Studies in related lysosomal storage disorders have examined the effects of missense mutations on enzyme processing and trafficking. Retention of misfolded protein in the ER is an important factor in specific variants that cause Gaucher disease 32, 33, 34, 35, Pompe disease 36 and GM1 gangliosidosis 37. In several cases, this insight has been used to examine if pharmacological chaperones, ERAD inhibitors and proteostasis regulators can rescue these misfolded variants and restore trafficking to the lysosome. Although these studies have provided some potential leads for therapeutic development, they have also highlighted that these approaches are often only successful with a limited subset of mutations potentially due to the severity of the misfolding defect (mentioned above). One of the current limitations in drug development for Krabbe disease is the lack of reliable high‐throughput (HT) approaches for the screening of new therapeutics against the wide range of mutations present in the patient population. Although activity measurements can be carried out in a HT manner 38, previous work has shown that GALC activity does not correlate well with disease severity and may not provide a good measure for treatment efficacy 25, 31. One of the primary reasons for this may be attributed to the fact that the activity assays are often conducted on whole cell lysates and so cannot distinguish between protein that is localized in the ER or the endocytic pathway leaving the question as to whether the active protein is still mislocalized unanswered. Recent work measuring the activity of samples enriched for the lysosomal fraction show a better correlation with disease severity providing a good first step towards more reliable activity measurements 31. Future development of the assays described here into a HT format would provide a complementary readout to these activity assays and confirm those conditions where GALC is successfully trafficked beyond the ER‐TGN and thus corrected for misfolding defects.

## Materials and Methods

### Cloning and mutagenesis of human GALC constructs

For large‐scale protein expression in mammalian cells wild‐type human GALC (hGALC) polymorphisms I546 and T546 were cloned into pSecTag2B vector with an N‐terminal His6 tag and stable HEK293T cell lines were established and cultured as described previously 3. For transient expression of mutants in mammalian tissue culture mutant hGALC constructs were generated in the pSecTag2b vector by Quikchange site directed mutagenesis of wild‐type hGALC in either the I546 or T546 background (as indicated). Constructs were then sub‐cloned into the pHLsec vector resulting in removal of the His tag and addition of a C‐terminal FLAG epitope tag.

### Expression and purification of human GALC protein

Large‐scale expression and IMAC‐affinity purification of hGALC proteins from HEK293T cells for antibody (Ab) generation, activity assays and thermal denaturation experiments was carried out as described previously for the equivalent mouse GALC constructs 21, 39.

### Monoclonal antibody generation

Monoclonal mouse anti‐human GALC antibodies were raised against full‐length secreted human GALC protein. Mice were immunized subcutaneously 2 times with 25 µg of purified human GALC, mixed with GERBU^®^ adjuvant. Before 3 days of fusion, the mice received an intravenous (i.v.) injection with 10 µg of antigen together with adrenalin. Spleen cells and SP2 myeloma cells were used for fusion. Positive clones were selected and cloned by repeated screening against the GALC protein using enzyme‐linked immunosorbent assay (ELISA). The performance and specificity of several antibody clones were validated using western blot and immunofluorescence microscopy against transfected cells.

### Cell culture

HEK293T and HeLa cells were maintained in Dulbecco's Modified Eagle Medium supplemented with 10% foetal bovine serum, 50 U/mL penicillin, 50 µg/mL streptomycin and 4 mm l‐glutamine. Cells were maintained at 37°C in humidified air with 5% CO_2_. HEK293T cells used for immunoblot studies were grown in Freestyle 293 expression medium and transiently transfected using Lipofectamine‐2000 transfection reagent. HeLa cells used for immunostaining were transiently transfected at 40% confluence using the TransIT‐HeLa Monster transfection reagent. For GALC uptake assays, serum‐free conditioned media from transfected or untransfected cells was harvested and filtered 72 h post‐transfection and then applied to untransfected cells for 48 h. Cells were lysed in 1% Triton X‐100 and subjected to immunoprecipitation using monoclonal mouse anti‐GALC antibody in the presence of protein A sepharose beads. GALC cellular uptake was detected by immunoblot, as described below.

### Immunoblotting and immunoprecipitation of GALC

Secreted protein was detected from conditioned media harvested 72 h post‐transfection. Cells were lysed in either 1% Triton X‐100 or 1% SDS in Tris‐buffered saline (TBS, 20 mm Tris–HCl, 150 mm NaCl, 5 mm MgCl_2_, 1 mm EDTA) in the presence of 10 mm N‐ethylmaleimide (NEM), 1 mm phenylmethylsulfonyl fluoride (PMSF) and protease inhibitors. Where appropriate, protein concentration was quantified via bicinchoninic acid (BCA) assay. For immunoprecipitation, 1000 µg cell lysate was precleared using IgG/protein A‐sepharose beads. Pre‐cleared lysate was incubated with anti‐FLAG or anti‐GALC antibodies in the presence of A‐sepharose beads and washed in TBS supplemented with 0.1% Triton X‐100. All samples were denatured and reduced in SDS loading buffer containing 20 mm DTT prior to SDS‐PAGE separation. For immunoblot studies, proteins were transferred to methanol‐activated Immobilon‐FL polyvinylidene fluoride (PVDF) membrane. After blocking, membranes were incubated with primary antibody followed by incubation with infrared fluorophore‐conjugated secondary antibody. Immuno‐reactive bands were visualised with an Odyssey infrared imager (LI‐COR Biosciences).

### Confocal immunofluorescence microscopy

Cells were grown on glass coverslips, washed with PBS and fixed in 4% PFA. Fixed cells were washed in PBS, quenched with 15 mm glycine and permeabilised in 0.1% saponin in PBS. Fixed cells were blocked in 1% BSA, 0.01% saponin in PBS before incubation with primary antibody diluted in 1% BSA with 0.01% saponin followed by fluorescent‐conjugated secondary antibody. Coverslips were mounted onto slides using mounting medium with DAPI (*Prolong Antifade*, Invitrogen Molecular Probes). Images were acquired on a Zeiss LSM880 confocal microscope with zeiss zen software. To analyse co‐localization, intensities were measured for ≥20 individual cells from ≥3 independent experiments and Pearson's correlation coefficients were calculated using the volocity 5.2 software (Perkin Elmer). Automatic Costes thresholds were applied to all images 40. Significance was calculated using two‐tailed *t*‐tests implemented in prism5 (graphpad), error bars represent SEM. Endogenous GALC is not detectable by immunostaining of untransfected HeLa cells (Figure S4A,B). HeLa cells transfected with the N‐terminally His‐tagged construct of GALC show equivalent cellular localization as the C‐terminally FLAG‐tagged construct (Figure S4, C).

### Antibodies

The antibodies used for immunostaining were: monoclonal mouse anti‐GALC clone mAb6 (described above), polyclonal rabbit anti‐cathepsin D (Calbiochem), polyclonal rabbit anti‐calreticulin (Pierce), polyclonal rabbit anti‐calnexin (Abcam), polyclonal rabbit anti‐REEP5 (proteintech), polyclonal goat AlexaFluor488‐conjugated anti‐mouse IgG (Life Technologies) and polyclonal goat AlexaFluor555‐conjugated anti‐rabbit IgG (Life Technologies). Antibodies used for immunoblotting were: monoclonal mouse anti‐FLAG (Sigma), monoclonal mouse anti‐GALC clones mAb1 and mAb2 (described above), polyclonal rabbit anti‐GALC (Abcam), polyclonal rabbit anti‐actin (Sigma), polyclonal goat IRdye680‐conjugated anti‐rabbit IgG (LiCor) and polyclonal goat IRdye800‐conjugated anti‐mouse IgG (LiCor).

### Deglycosylation of GALC

Conditioned media and cells lysed in 1% SDS (as described above) were denatured at 100°C for 10 min and were either subjected to EndoH or PNGaseF treatment (NEB) for 4 h at 37°C.

### Enzyme activity assays

Activity assays were carried out as described previously 21. In brief, activity assays were carried out at 37°C using purified hGALC in 20 mm sodium acetate, 150 mm NaCl, 0.1% v/v NP‐40, pH 4.6 and chromogenic substrate 4‐nitrophenyl‐β‐d‐galactopyranoside (4NβDG). Product 4‐nitrophenol was monitored by absorbance at 410 nm following addition of stopping buffer (360 mm NaOH, 280 mm glycine, pH 10.6). *K*_m_ and *V*_max_ were obtained from plots of initial velocity against substrate concentration by non‐linear curve‐fitting to the Michaelis–Menten equation using prism5 (graph pad). *k*_cat_ was determined as the gradient of the linear plot of *V*_max_ against enzyme concentration.

### Differential scanning fluorimetry (DSF)

DSF experiments were carried out as described previously 39. In brief, 5 µg of purified hGALC in PBS, pH 7.4 was combined with 5× SyPRO Orange dye prior to thermal denaturation performed using a Bio‐Rad MiniOpticon RT‐PCR thermal cycler. The melting temperature (*T*_m_) was the inflexion point of the sigmoidal curve obtained by curve fitting using DSF analysis scripts and prism5 (graph pad).

## Supporting information

Editorial Process

**Table S1: Missense mutations of GALC described in the text**. This table is first associated with Figure 1 but is relevant to the whole paper and describes the alternative numbering used in the literature allowing for greater clarity when comparing with other published data using this numbering scheme.
**Table S2: Enzyme activity of WT GALC I546 and the T546 polymorphism**. This table is associated with Figure 6 and provides the details of the enzyme activity values K_m_, V_max_ and k_cat_ calculated from the data in Figure 6.
**Table S3: Proportion of side chain buried in the structure, prediction and identified defect**. This table is associated with the Discussion and summarises the correlation between the buried surface area of a side chain as calculated from the structure and the predicted effect this would have on the protein compared with the molecular mechanism identified in this work.
**Figure S1: Colocalization of T513M and L618S missense mutations with additional ER markers**. This figure provides additional representative confocal images of T513M and L618S colocalized with the ER markers calnexin and REEP5. These images are associated with Figure 3.
**Figure S2: Colocalization of additional missense mutations of GALC with ER markers**. This figure provides representative confocal images of additional missense mutations I583S, R515H and Y319C showing colocalization with the ER markers calreticulin and calnexin. These images are associated with Figure 3.
**Figure S3: Effect of polymorphic background on trafficking of G270D and Y551S**. This figure provides additional representative confocal images of G270D and Y551S in both polymorphic backgrounds colocalized with the ER marker REEP5. These images are associated with Figure 7.
**Figure S4: Representative confocal images from untransfected HeLa cells and cells expressing N‐terminally tagged GALC constructs**. This figure provides evidence that endogenous GALC is not detectable in untransfected HeLa cells and that a construct of GALC possessing a tag at the N‐terminus displays similar cellular localization as the C‐terminally tagged construct. This figure is associated with the Materials and Methods section.
