# Supplementary material for: Molecular Mechanisms of Disease Pathogenesis Differ in Krabbe Disease Variants
Source: Traffic. 2016 May 30;17(8):908–22. doi: 10.1111/tra.12404 (PMC4949656; doi:10.1111/tra.12404)
Supplement: Supplementary file 1 — Editorial Process [file TRA-17-908-s001.docx]

| Title: | **Molecular Mechanisms of Disease Pathogenesis Differ in Krabbe Disease Variants** |
| --- | --- |
| Authors: | Samantha J. Spratley, Chris H. Hill, Agnete H. Viuff, James R. Edgar, Karsten Skjødt and Janet E. Deane |
| Article Type: | Original Research |
|  |  |
| Monitoring Editor | Mickey Marks |
| Date Submitted | 3 March 2016 |
| Date for Decision 1 | 9 March 2016 |
| Date Resubmitted | 18 March 2016 |
| Date for Decision 2 | 18 March 2016 |
| Date Resubmitted | 7 April 2016 |
| Date for Decision 3 |  |
| Accepted |  |

**Decision and Reviews**

Dear Janet,

Thank you for submitting your manuscript “Molecular Mechanisms of Disease Pathogenesis Differ in Krabbe Disease Variants” to Traffic. I asked a colleague who is an expert in the field to review this paper and his/her verbatim comments are appended below. I share the enthusiasm of the referee for the work presented in this paper and agree that it will be of interest to the readers of Traffic. However, there are a few issues that need to be addressed before the paper can be accepted for publication. Specifically, I agree with the referee that you will need to provide additional support for the localization of some of your mutants to the ER, and that REEP5 is a questionable marker (to my eye, the pattern of your GALC mutants in Figure 3 looks more like ER than the "ER marker" does). The other issues raised by the referee can all be addressed by revision of the text or clarification of the figures.

Although I cannot accept your manuscript for publication at this point, I believe that you will be able to quickly address the referee's concerns and I look forward to receiving your revised manuscript. To expedite handling when you resubmit please be sure to include a response outlining how you have addressed each of the referee's concerns.

Sincerely,

Mickey Marks, Ph.D.
Co-Editor

________________________________________________________

Referee's Comments to the Authors


Referee: 1

Comments to the Author
The manuscript by Spratley et al. describes the characterization and classification of missense mutations of GALC, the enzyme underlying Krabbe disease, in order to define which mutations cause a misfolded enzyme and which affect traffic into lysosomes. The experiments are mostly well designed and executed and the conclusions drawn are appropriate and supported by the data. The paper advances the understanding of Krabbe disease and impacts other lysosomal storage diseases. Particularly illuminating are the analyses of the hyper-glycosylated mutation and of the effect of the polymorphic sequence background on trafficking mutants (Figs. 5-7).

One major experimental issue does require more data - the identification of the ER as the compartment where some of the mutants are trapped. While the co-localization with cathepsin D in Figs. 3, 4, S1 is convincing, the co-localization in the ER is not. In several panels, the pattern is not reticular and the quality of co-localization is inferior to the lysosomal co-localization. The lack of obvious ER pattern with even with the wild type construct (Fig. 3) indicates either that the ER pool of GALC is not dominant at steady state, or alternatively - that the ER marker used is not reliable. I think that the problem is due in part to the use of REEP5 as an ER marker. REEP5 is not a commonly used ER marker and the authors should better justify its use. More convincing will be to supplement the use of REEP5 by co-localization of GALC with one of the many canonical markers that are resident (almost) only in the ER. If the co-localization result is similar, the compartmental distribution of GALC should be discussed, as it is not necessarily what is commonly expected.

A second issue is of interpretation. In the Discussion, the authors state that “Specifically, those mutations that cause misfolding, giving rise to lack of secretion, processing and primarily ER co-localization, should be targeted by pharmacological chaperone therapies while those that retain significant capacity for correct trafficking and processing will require future enzyme replacement approaches.” However, it can be argued that many mutations that cause misfolding maybe too catastrophic to be repaired and thus they would require enzyme replacement approaches, while milder mutations that retain significant secretion would benefit more from improvement by chemical chaperones.

Lastly, I find that the current manuscript would be more scholastic and appeal to a wider readership if its scope was broadened to include comparisons to mutants in other lysosomal storage diseases; the classification of mutations is similar in many of those and lessons from treating other defective enzymes would be applicable to GALC.

More Minor Comments:

Expression – from the M&M it is unclear whether the clones expressed in mammalian cells have both a His tag and a FLAG tag, or whether the two tags are exclusive of each other. In any case, is there data showing that the C terminal tags, which evidently are in the domain of the protein that is processed upon traffic to the lysosomes, do not affect the traffic?

Fig 1 – generally agree with the interpretation, though the smeared endo H treated form in the sup indicates that all the variants are not completely resistant to endo H
The uniformity of the variants is still impressive (and surprising)

Fig 1 needs MW markers

In Fig 3 – the antibody that was used to detect GALC in this experiment should be specified in the legend, not just in the Results.
Using the new monoclonal antibodies, is there no/low expression of GALC in 293T cells, and if so, how does the expression of the exogenous constructs impact the expression of the endogenous GALC?

The gel resolution in 5B is great. The authors should refer to this pattern when they discuss the normal glycosylation pattern of GALC at the beginning of Results.
The experiment in Figure 5 is very convincing.

Figure S1 does not show missense mutations that are trapped in the ER; rather, if shows mutations that do not co-localize with cathepsin D.

_____________________________________________________________________________**Author Rebuttal**

**18 March 2016**

We thank the referee and the Co-Editor for their helpful comments. Below are details of how we have addressed each of the referee’s concerns. A pdf version of these responses has also been uploaded that contains the figures mentioned in this text.

Referee: 1

Referee: One major experimental issue does require more data - the identification of the ER as the compartment where some of the mutants are trapped. While the co-localization with cathepsin D in Figs. 3, 4, S1 is convincing, the co-localization in the ER is not. In several panels, the pattern is not reticular and the quality of co-localization is inferior to the lysosomal co-localization. The lack of obvious ER pattern with even with the wild type construct (Fig. 3) indicates either that the ER pool of GALC is not dominant at steady state, or alternatively - that the ER marker used is not reliable. I think that the problem is due in part to the use of REEP5 as an ER marker. REEP5 is not a commonly used ER marker and the authors should better justify its use. More convincing will be to supplement the use of REEP5 by co-localization of GALC with one of the many canonical markers that are resident (almost) only in the ER. If the co-localization result is similar, the compartmental distribution of GALC should be discussed, as it is not necessarily what is commonly expected.

Response: REEP5 was selected as our ER marker as it proved to be more reproducible in our hands than the available calreticulin and calnexin antibodies we initially tested. The REEP5 antibody from proteintech had been used in a number of recent publications and was identified as an ER-resident protein (1-4).

However, we agree with the reviewer that additional evidence of ER localization may be more convincing and have carried out additional immunofluorescence experiments with alternative calreticulin and calnexin antibodies and provided two additional figures to supplement the use of REEP5. These figures have been included as new supporting Figures S1 and S2 and illustrate co-localization of T513M, L618S, I583S, R515H and Y319C with both calreticulin and calnexin. We think this additional supporting information provides clear evidence for our statements that these GALC variants are indeed trapped in the ER. These additional figures are included at the end of this document for clarity.

Referee: A second issue is of interpretation. In the Discussion, the authors state that “Specifically, those mutations that cause misfolding, giving rise to lack of secretion, processing and primarily ER co-localization, should be targeted by pharmacological chaperone therapies while those that retain significant capacity for correct trafficking and processing will require future enzyme replacement approaches.” However, it can be argued that many mutations that cause misfolding maybe too catastrophic to be repaired and thus they would require enzyme replacement approaches, while milder mutations that retain significant secretion would benefit more from improvement by chemical chaperones.

Response: We agree with the referee’s point and have modified the Discussion to address this directly. The new text reads: “Mutations that cause misfolding, giving rise to lack of secretion, processing and primarily ER co-localization, may be good targets for pharmacological chaperone therapies while those that retain significant capacity for correct trafficking and processing will require future enzyme replacement approaches. However, the extent of misfolding caused by specific mutations will alter the effectiveness of pharmacological chaperones. Specifically, the misfolding caused by some mutations may prove too severe to respond to these approaches and will therefore require enzyme replacement strategies.”

Referee: Lastly, I find that the current manuscript would be more scholastic and appeal to a wider readership if its scope was broadened to include comparisons to mutants in other lysosomal storage diseases; the classification of mutations is similar in many of those and lessons from treating other defective enzymes would be applicable to GALC.

Response: We have added a section to the discussion (included below) referencing other lysosomal storage diseases where similar studies have been carried out. We have restricted this to those papers that provide some microscopy data to directly support the claim of ER localization. We also mention the work describing use of small molecules to rescue these defects. This discussion then leads into the last section of the discussion describing the need for HT approaches.

“Studies in related lysosomal storage disorders have examined the effects of missense mutations on enzyme processing and trafficking. Retention of misfolded protein in the ER is an important factor in specific variants that cause Gaucher disease (32-35), Pompe disease (36) and GM1 gangliosidosis (37). In several cases, this insight has been used to examine if pharmacological chaperones, ERAD inhibitors and proteostasis regulators can rescue these misfolded variants and restore trafficking to the lysosome. Although these studies have provided some potential leads for therapeutic development, they have also highlighted that these approaches are often only successful with a limited subset of mutations potentially due to the severity of the misfolding defect (mentioned above).”

More Minor Comments:

Referee: Expression – from the M&M it is unclear whether the clones expressed in mammalian cells have both a His tag and a FLAG tag, or whether the two tags are exclusive of each other. In any case, is there data showing that the C terminal tags, which evidently are in the domain of the protein that is processed upon traffic to the lysosomes, do not affect the traffic?

Response: We have rewritten the text of this section of the M&M to clarify the details of the tags used in each construct. Also we apologize that we had incorrectly annotated the pSecTag2B constructs as having a C-terminal tag when in fact it is an N-terminal His tag (as detailed in our previous publications using similar constructs). The FLAG tag in the pHLSec vector is at the C-terminus of the protein and so these two constructs represent versions of GALC with tags at opposite ends. The domain of the protein that is processed upon traffic to the lysosomes is actually the β-sandwich domain (residues 338-452) not the C-terminal lectin domain (residues 472-668) and so it is unlikely that the tags would interfere with processing. However, to check that this is indeed the case we have carried out an additional immunofluorescence microscopy experiment with the N-terminal His-tagged construct to confirm co-localization with cathepsin D similar to that seen with the C-terminally FLAG-tagged constructs. Below are three representative merged images showing immunostaining similar to WT as shown in Fig 3.

Referee: Fig 1 – generally agree with the interpretation, though the smeared endo H treated form in the sup indicates that all the variants are not completely resistant to endo H. The uniformity of the variants is still impressive (and surprising)

Response: We have added the word primarily to the sentence describing the EndoH resisitance such that it now reads: “…while the protein secreted into the media was primarily EndoH resistant…”

Referee: Fig 1 needs MW markers

Response: These have now been added.

Referee: In Fig 3 – the antibody that was used to detect GALC in this experiment should be specified in the legend, not just in the Results.

Response: We have now specified in the legend that it was the monoclonal antibody against GALC that was used in this experiment. We have only added this to the first legend (Fig 3) rather than repeat it in subsequent relevant figure legends but leave it to the discretion of the editor as to whether this should be explicitly stated also in the legends for Figs 4, 5 and 7.

Referee: Using the new monoclonal antibodies, is there no/low expression of GALC in 293T cells, and if so, how does the expression of the exogenous constructs impact the expression of the endogenous GALC?

Response: There is no detectable GALC expression in HEK293T or HeLa cells either following IP-WB from cells or using the monoclonal antibodies in immunofluorescence (IF) microscopy. In several panels in the manuscript there are images showing individual cells that have not been transfected, illustrating that no endogenous GALC is detectable (left 3 panels below). Images of untransfected cells taken in an equivalent way to those included in the manuscript show no staining. We have taken additional images of untransfected cells with significantly higher laser power and have included a representative image below (right 2 panels). We interpret these images as non-specific staining only. As we cannot detect endogenous GALC by either IP or IF in HeLa or HEK293T cells we cannot comment on the effect of exogenous constructs on this expression.

Referee: The gel resolution in 5B is great. The authors should refer to this pattern when they discuss the normal glycosylation pattern of GALC at the beginning of Results. The experiment in Figure 5 is very convincing.

Response: We have added a reference to Fig. 5B at the beginning of the results (p4) when we first mention the glycosylation of GALC.

Referee: Figure S1 does not show missense mutations that are trapped in the ER; rather, if shows mutations that do not co-localize with cathepsin D.

Response: This figure has been updated with new images of co-localization with alternative ER markers as part of the response to the first comment above.


References

1. Papadopoulos C, Orso G, Mancuso G, Herholz M, Gumeni S, Tadepalle N, Jungst C, Tzschichholz A, Schauss A, Honing S, Trifunovic A, Daga A, Rugarli EI. Spastin binds to lipid droplets and affects lipid metabolism. PLoS Genet 2015;11:e1005149.

2. Galea G, Bexiga MG, Panarella A, O'Neill ED, Simpson JC. A high-content screening microscopy approach to dissect the role of Rab proteins in Golgi-to-ER retrograde trafficking. J Cell Sci 2015;128:2339-2349.

3. Ulengin I, Park JJ, Lee TH. ER network formation and membrane fusion by atlastin1/SPG3A disease variants. Mol Biol Cell 2015;26:1616-1628.

4. Chang J, Lee S, Blackstone C. Protrudin binds atlastins and endoplasmic reticulum-shaping proteins and regulates network formation. Proc Natl Acad Sci U S A 2013;110:14954-14959.

 
Supplementary Figure S1

Figure S1. Co-localization of missense mutations of GALC with the ER marker calreticulin. Representative confocal images of HeLa cells transfected with wild-type and Krabbe disease mutations T513M, L618S, I583S, R515H and Y319C. Cells were plated onto glass coverslips, fixed and immunostained using monoclonal antibody against GALC (green) and the ER marker calreticulin (red). Nuclei were stained with DNA-binding dye, DAPI (blue). Scale bar 10 µm.
 
Supplementary Figure S2

Figure S2. Co-localization of missense mutations of GALC with the ER marker calnexin. Representative confocal images of HeLa cells transfected with wild-type and Krabbe disease mutations T513M, L618S, I583S, R515H and Y319C. Cells were plated onto glass coverslips, fixed and immunostained using monoclonal antibody against GALC (green) and the ER marker calnexin (red). Nuclei were stained with DNA-binding dye, DAPI (blue). Scale bar 10 µm.

_____________________________________________________________________________**Decision and Reviews**

**Date 18 March 2016**

Dear Janet,

Thank you for submitting your revised manuscript, "Molecular Mechanisms of Disease Pathogenesis Differ in Krabbe Disease Variants", for publication in Traffic. Your response to the original review was lightning fast! I agree that your response includes valid answers to all of the concerns raised by the reviewer, and the additional data you provided are compelling. However, I think it might be worth your while to spend a couple of days to more comprehensively incorporate these data into the manuscript. In particular, please consider the following:
• It is awesome that you were able to provide the colocalization data with calnexin and calreticulin so quickly, and the data in Suppl. Figs. 1 and 2 look great. As I predicted in the previous decision letter, these supplementary data look considerably more convincing than those in the main Figures 3 and 4, in which REEP5 does not look to be as robust a pan-ER marker as your cited publications suggest (this is not uncommon - we had similar issues in the past with a melanosomal protein). The lack of overlap in Figure 3 definitely drew raised eyebrows from both the reviewer and myself. Given the new images that you provide, I would also imagine that the Pearson's Correlation coefficient for overlap of the T513M and L618S mutants with calreticulin would be substantially higher than that obtained with REEP5. I wonder if you would be best served to consider replacing the less convincing data in Figure 3 with the more convincing data of Suppl. Fig. S1, adding the new PCC, and relegating the less convincing data to the Supplement. I will not make this a requirement for acceptance, but please consider it an encouraging nudge!
• The control images that you provided in the Response to the Reviewer (localization of N-terminally His-tagged GALC to lysosomes on page 2 of your rebuttal, and lack of GALC expression in untransfected HeLa or HEK293T cells in the left three panels on page 3 of your rebuttal) seem to me to be important ones to validate that your reagents are what they are supposed to be. As for the latter, I did not see an untransfected cell in any of the Figures in the paper except perhaps for a corner of a cell jutting into a frame or two; you did quite a nice job of focusing on single cells in these panels. I thus urge you to consider adding these figures from the Response to Reviewers to the manuscript as supplementary data, perhaps cited from the Materials and Methods section together with the explanations that you provide in the Response.
• Regarding the GALC antibodies used in different experiments, if the same antibody is used throughout the manuscript for the immunofluorescence microscopy and a different antibody is used throughout the manuscript for immunoblotting, I would state this explicitly in the Materials and Methods - then you don't have to worry about restating it each time in the Figure Legends.
With these changes, I would be pleased to accept your manuscript for publication in Traffic. Congratulations on a job well (and speedily) done!

Yours,

Mickey Marks
Co-Editor

_____________________________________________________________________________**Author Rebuttal**

**Date 10 April 2016**

Below is a modified version of our previous response addressing the additional requests of 18th March 2016.

Response to Referee’s Comments

We thank the referee and the Co-Editor for their helpful comments. Below are details of how we have addressed each of the referee’s concerns.

Referee: 1

One major experimental issue does require more data - the identification of the ER as the compartment where some of the mutants are trapped. While the co-localization with cathepsin D in Figs. 3, 4, S1 is convincing, the co-localization in the ER is not. In several panels, the pattern is not reticular and the quality of co-localization is inferior to the lysosomal co-localization. The lack of obvious ER pattern with even with the wild type construct (Fig. 3) indicates either that the ER pool of GALC is not dominant at steady state, or alternatively - that the ER marker used is not reliable. I think that the problem is due in part to the use of REEP5 as an ER marker. REEP5 is not a commonly used ER marker and the authors should better justify its use. More convincing will be to supplement the use of REEP5 by co-localization of GALC with one of the many canonical markers that are resident (almost) only in the ER. If the co-localization result is similar, the compartmental distribution of GALC should be discussed, as it is not necessarily what is commonly expected.

REEP5 was selected as our ER marker as it proved to be more reproducible in our hands than the available calreticulin and calnexin antibodies we initially tested. The REEP5 antibody from proteintech had been used in a number of recent publications and was identified as an ER-resident protein (1-4).

However, we agree with the reviewer that additional evidence of ER localization may be more convincing and have carried out additional immunofluorescence experiments with alternative calreticulin and calnexin antibodies. These new data provide more compelling evidence for the co-localization of the misfolded variants with the ER markers. We have replaced all the REEP5 panels in the main paper with images of co-localization with calreticulin (new Fig. 3 and Fig. 4). We have also calculated new Pearson’s correlation coefficients with the calreticulin data and although these do not change the interpretation of the effects of these mutations they do illustrate better co-localization than with the REEP5. We have moved the REEP5 images to the supplementary information along with additional images of the co-localization with another ER marker calnexin (Fig S1). Using these new ER markers (calreticulin and calnexin) we have also confirmed the ER localization of the additional misfolded mutants I583S, R515H and Y319C (Fig. S2). We think this additional supporting information provides clear evidence for our statements that these GALC variants are indeed trapped in the ER.

A second issue is of interpretation. In the Discussion, the authors state that “Specifically, those mutations that cause misfolding, giving rise to lack of secretion, processing and primarily ER co-localization, should be targeted by pharmacological chaperone therapies while those that retain significant capacity for correct trafficking and processing will require future enzyme replacement approaches.” However, it can be argued that many mutations that cause misfolding maybe too catastrophic to be repaired and thus they would require enzyme replacement approaches, while milder mutations that retain significant secretion would benefit more from improvement by chemical chaperones.

We agree with the referee’s point and have modified the Discussion to address this directly. The new text reads: “Mutations that cause misfolding, giving rise to lack of secretion, processing and primarily ER co-localization, may be good targets for pharmacological chaperone therapies while those that retain significant capacity for correct trafficking and processing will require future enzyme replacement approaches. However, the extent of misfolding caused by specific mutations will alter the effectiveness of pharmacological chaperones. Specifically, the misfolding caused by some mutations may prove too severe to respond to these approaches and will therefore require enzyme replacement strategies.”

Lastly, I find that the current manuscript would be more scholastic and appeal to a wider readership if its scope was broadened to include comparisons to mutants in other lysosomal storage diseases; the classification of mutations is similar in many of those and lessons from treating other defective enzymes would be applicable to GALC.

We have added a section to the discussion (included below) referencing other lysosomal storage diseases where similar studies have been carried out. We have restricted this to those papers that provide some microscopy data to directly support the claim of ER localization. We also mention the work describing use of small molecules to rescue these defects. This discussion then leads into the last section of the discussion describing the need for HT approaches.

“Studies in related lysosomal storage disorders have examined the effects of missense mutations on enzyme processing and trafficking. Retention of misfolded protein in the ER is an important factor in specific variants that cause Gaucher disease (32-35), Pompe disease (36) and GM1 gangliosidosis (37). In several cases, this insight has been used to examine if pharmacological chaperones, ERAD inhibitors and proteostasis regulators can rescue these misfolded variants and restore trafficking to the lysosome. Although these studies have provided some potential leads for therapeutic development, they have also highlighted that these approaches are often only successful with a limited subset of mutations potentially due to the severity of the misfolding defect (mentioned above).”

More Minor Comments:

Expression – from the M&M it is unclear whether the clones expressed in mammalian cells have both a His tag and a FLAG tag, or whether the two tags are exclusive of each other. In any case, is there data showing that the C terminal tags, which evidently are in the domain of the protein that is processed upon traffic to the lysosomes, do not affect the traffic?

We have rewritten the text of this section of the M&M to clarify the details of the tags used in each construct. Also we apologize that we had incorrectly annotated the pSecTag2B constructs as having a C-terminal tag when in fact it is an N-terminal His tag (as detailed in our previous publications using similar constructs). The FLAG tag in the pHLSec vector is at the C-terminus of the protein and so these two constructs represent versions of GALC with tags at opposite ends. The domain of the protein that is processed upon traffic to the lysosomes is actually the β-sandwich domain (residues 338-452) not the C-terminal lectin domain (residues 472-668) and so it is unlikely that the tags would interfere with processing. However, to check that this is indeed the case we have carried out an additional immunofluorescence microscopy experiment with the N-terminal His-tagged construct to confirm co-localization with cathepsin D similar to that seen with the C-terminally FLAG-tagged constructs. We have included representative confocal images of the co-localization of N-terminally tagged GALC with cathepsin D as part of an additional supporting figure (Fig. S4) and have referenced this from the Materials and Methods section.


Fig 1 – generally agree with the interpretation, though the smeared endo H treated form in the sup indicates that all the variants are not completely resistant to endo H. The uniformity of the variants is still impressive (and surprising)

We have added the word primarily to the sentence describing the EndoH resisitance such that it now reads: “…while the protein secreted into the media was primarily EndoH resistant…”

Fig 1 needs MW markers

These have now been added.

In Fig 3 – the antibody that was used to detect GALC in this experiment should be specified in the legend, not just in the Results.

We have now specified in the legend that it was the monoclonal antibody against GALC that was used in this experiment. Different monoclonal antibodies were used for the IPs and the confocal microscopy so we have specifically labelled these in the Methods section and in figure legends where appropriate.

Using the new monoclonal antibodies, is there no/low expression of GALC in 293T cells, and if so, how does the expression of the exogenous constructs impact the expression of the endogenous GALC?

There is no detectable GALC expression in HEK293T or HeLa cells either following IP-WB from cells or using the monoclonal antibodies in immunofluorescence (IF) microscopy. We have included additional images as a supporting figure (Fig. S4) showing individual cells that have not been transfected, illustrating no endogenous GALC is detectable. Images of untransfected cells taken in an equivalent way to those included in the manuscript show no staining. We have taken additional images of untransfected cells with significantly higher laser power and have also included these in Fig. S4. We interpret these images as non-specific staining only. As we cannot detect endogenous GALC by either IP or IF in HeLa or HEK293T cells we cannot comment on the effect of exogenous constructs on this expression.

The gel resolution in 5B is great. The authors should refer to this pattern when they discuss the normal glycosylation pattern of GALC at the beginning of Results. The experiment in Figure 5 is very convincing.

We have added a reference to Fig. 5B at the beginning of the results (p4) when we first mention the glycosylation of GALC.

Figure S1 does not show missense mutations that are trapped in the ER; rather, if shows mutations that do not co-localize with cathepsin D.

This figure has been updated with new images of co-localization with alternative ER markers as part of the response to the first comment above.


References

1. Papadopoulos C, Orso G, Mancuso G, Herholz M, Gumeni S, Tadepalle N, Jungst C, Tzschichholz A, Schauss A, Honing S, Trifunovic A, Daga A, Rugarli EI. Spastin binds to lipid droplets and affects lipid metabolism. PLoS Genet 2015;11:e1005149.

2. Galea G, Bexiga MG, Panarella A, O'Neill ED, Simpson JC. A high-content screening microscopy approach to dissect the role of Rab proteins in Golgi-to-ER retrograde trafficking. J Cell Sci 2015;128:2339-2349.

3. Ulengin I, Park JJ, Lee TH. ER network formation and membrane fusion by atlastin1/SPG3A disease variants. Mol Biol Cell 2015;26:1616-1628.

4. Chang J, Lee S, Blackstone C. Protrudin binds atlastins and endoplasmic reticulum-shaping proteins and regulates network formation. Proc Natl Acad Sci U S A 2013;110:14954-14959.

_____________________________________________________________________________**Decision and Reviews Date**

_____________________________________________________________________________**Author Rebuttal Date**

_____________________________________________________________________________**Accepted and Sent to Press Date**
