# Supplementary material for: Molecular Mechanisms of Disease Pathogenesis Differ in Krabbe Disease Variants
Source: Traffic. 2016 May 30;17(8):908–22. doi: 10.1111/tra.12404 (PMC4949656; doi:10.1111/tra.12404)
Supplement: Supplementary file 2 — Table S1: Missense mutations of GALC described in the text. This table is first associated with Figure 1 but is relevant to the whole paper and describes the alternative numbering used in the literature allowing for greater clarity when comparing with other published data using this numbering scheme. Table S2: Enzyme activity of WT GALC I546 and the T546 polymorphism. This table is associated with Figure 6 and provides the details of the enzyme activity values Km, V max and k cat calculated from the data in Figure 6. Table S3: Proportion of side chain buried in the structure, prediction and identified defect. This table is associated with the Discussion and summarises the correlation between the buried surface area of a side chain as calculated from the structure and the predicted effect this would have on the protein compared with the molecular mechanism identified in this work. Figure S1: Colocalization of T513M and L618S missense mutations with additional ER markers. This figure provides additional representative confocal images of T513M and L618S colocalized with the ER markers calnexin and REEP5. These images are associated with Figure 3. Figure S2: Colocalization of additional missense mutations of GALC with ER markers. This figure provides representative confocal images of additional missense mutations I583S, R515H and Y319C showing colocalization with the ER markers calreticulin and calnexin. These images are associated with Figure 3. Figure S3: Effect of polymorphic background on trafficking of G270D and Y551S. This figure provides additional representative confocal images of G270D and Y551S in both polymorphic backgrounds colocalized with the ER marker REEP5. These images are associated with Figure 7. Figure S4: Representative confocal images from untransfected HeLa cells and cells expressing N‐terminally tagged GALC constructs. This figure provides evidence that endogenous GALC is not detectable in untransfected HeLa cells and that a construct of GALC pos [file TRA-17-908-s002.pdf]

SUPPORTING INFORMATION

Supplementary Table S1. Missense mutations of GALC described in the text

Mutation	Alternate numbering *
G41S	G57S
R63H	R79H
E215K	E231K
G270D	G286D
N279T	N295T
P302R	P318R
Y319C	Y335C
R380W/L	R396W/L
T513M	T529M
R515H	R531H
I546T §	I562T
Y551S	Y567S
I583S	I599S
L618S	L634S

* The first start site in exon 1 is used for numbering in Uniprot entry P54803 and is has also been adopted by some publications. For clarity, these designations are also provided.

§ Polymorphism, also annotated as 1637T>C

Table S2. Enzyme activity of WT GALC I546 and the T546 polymorphism

Enzyme	K_m (mM)	V_{max} (nmol min^{-1} µg^{-1})	k_{cat} (s^{-1})	k_{cat}/K_m (s^{-1} M^{-1})
GALC – I546	3.3 ± 0.06	9.87 ± 0.2	11.9	3,600
GALC – T546	3.6 ± 0.13	8.74 ± 0.3	10.6	2,900

Table S3. Proportion of side chain buried in the structure, prediction and identified defect

Mutation	% Buried ‡	Structure-based Prediction	Secreted	Localization	Defect
G41S	100	Misfolded	No		Misfolded
G270D	100	Misfolded	I546 dependent	I546 dependent	Subtle misfolded
T513M	100	Misfolded	No	ER	Misfolded
I546T §	100	Misfolded	No	Lysosomal	Subtle misfolded
I583S	100	Misfolded	No	ER	Misfolded
L618S	100	Misfolded	No	ER	Misfolded
N279T	99	Misfolded	Yes	ER	Hyper-glycosylated Not endocytosed
Y319C	99	Misfolded	No	ER	Misfolded
P302R	92	Misfolded	Yes		Not misfolded
R515H	82	Uncertain	No	ER	Misfolded
R63H	79	Uncertain	No		Misfolded
Y551S	78	Uncertain	No	ER	Misfolded
E215K	57	Not misfolded	Yes	Lysosomal	Not misfolded
R380W/L	48	Not misfolded	Yes	Lysosomal	Not misfolded Catalytic defect

‡ Using the mouse GALC structure (PDB 3ZR5) the proportion of each sidechain that is buried in the structure is reported as a percentage of that residue's entire surface area

§ Polymorphism, not disease-causing

Supplementary Figure S1

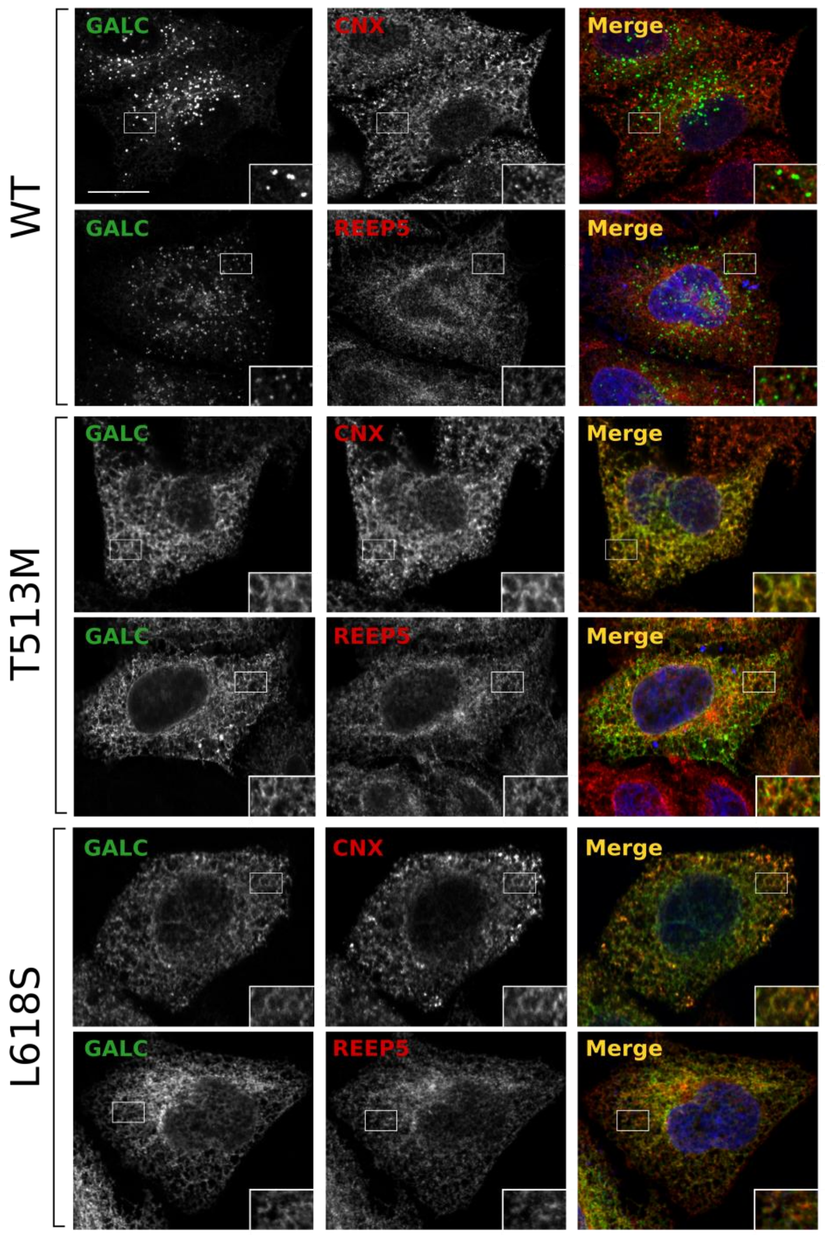

Figure S1. Co-localization of T513M and L618S missense mutations with additional ER markers. Representative confocal images of HeLa cells transfected with wild-type and Krabbe disease mutations T513M and L618S. Cells were plated onto glass coverslips, fixed and immunostained using monoclonal antibody against GALC (green) and the ER markers calnexin (CNX, red) or REEP5 (red). Nuclei were stained with DNA-binding dye, DAPI (blue). Scale bar 10 µm.

Supplementary Figure S2

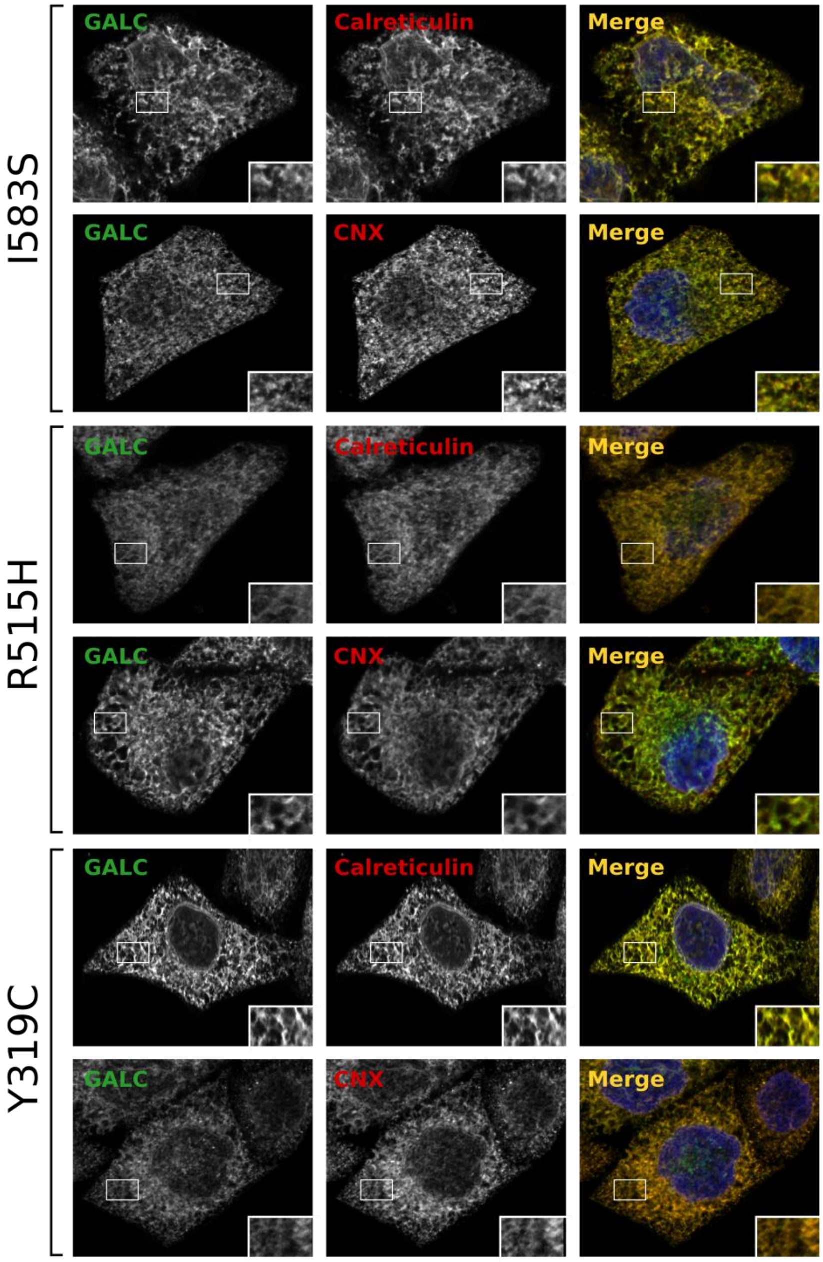

Figure S2. Co-localization of additional missense mutations of GALC with ER markers. Representative confocal images of HeLa cells transfected with wild-type and Krabbe disease mutations I583S, R515H and Y319C. Cells were plated onto glass coverslips, fixed and immunostained using monoclonal antibody against GALC (green) and the ER markers calreticulin (red) or calnexin (CNX, red). Nuclei were stained with DNA-binding dye, DAPI (blue). Scale bar 10 µm.

Supplementary Figure S3

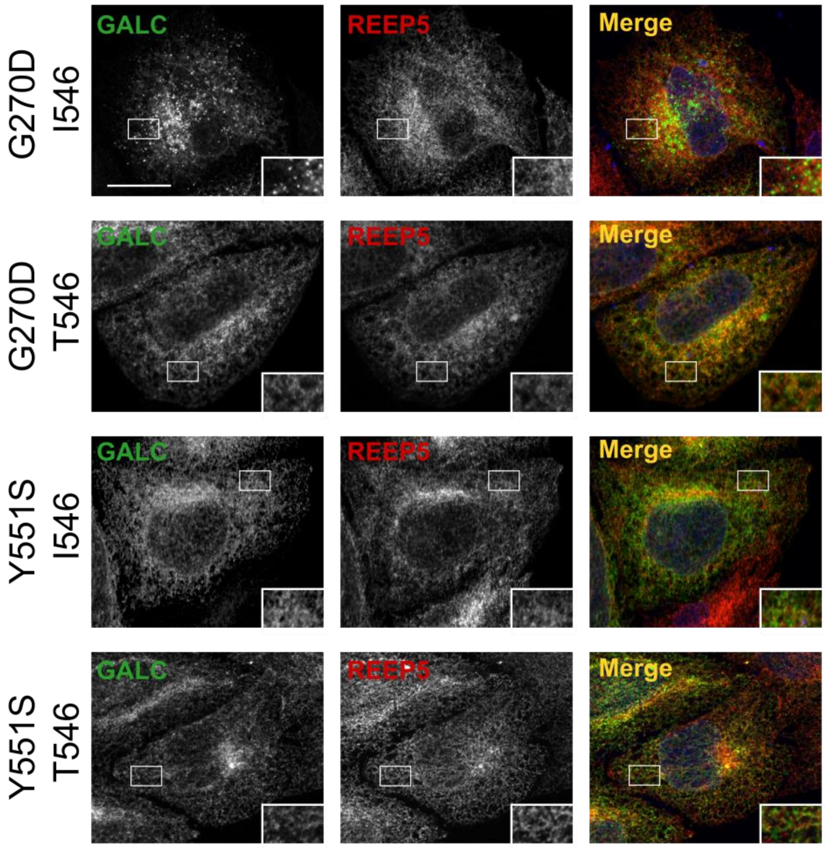

Figure S3. Effect of polymorphic background on trafficking of G270D and Y551S. Representative confocal images of HeLa cells transfected with Krabbe mutations G270D and Y551S in polymorphic backgrounds I546 and T546. Cells were plated onto glass coverslips, fixed and immunostained for GALC (green), the ER marker REEP5 (red). Nuclei were stained with DNA-binding dye, DAPI (blue). Scale bar 10 µm.

Supplementary Figure S4

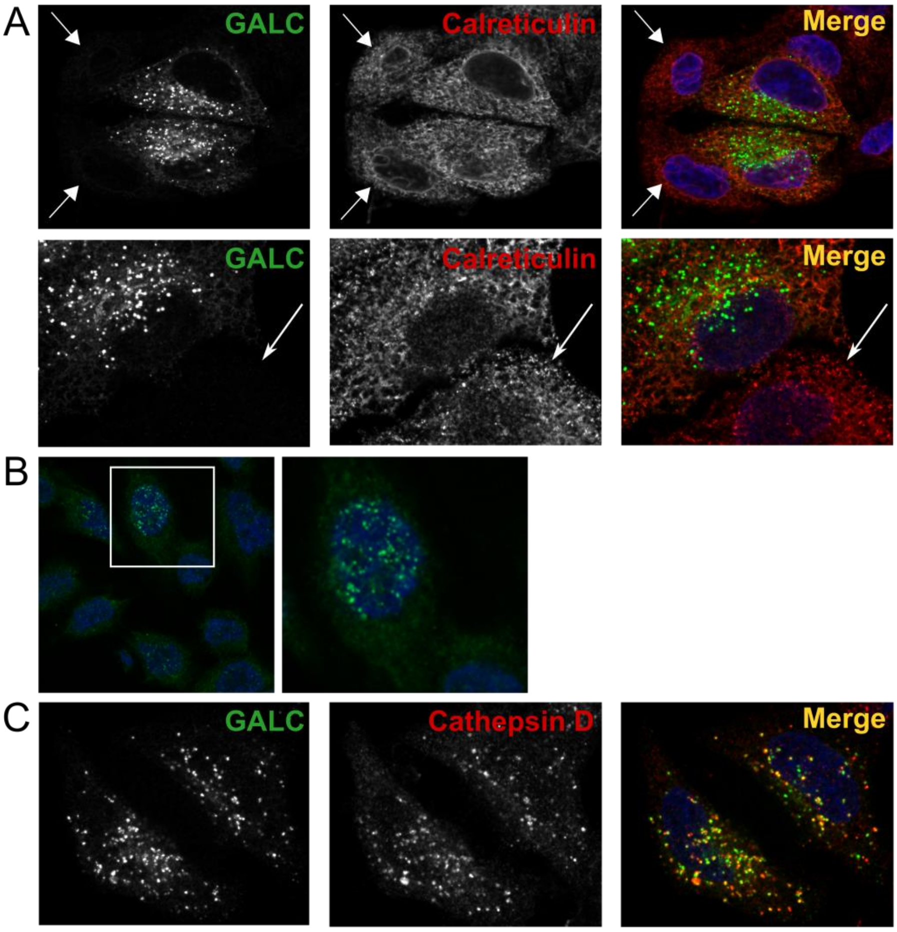

Figure S4. Representative confocal images from untransfected HeLa cells and cells expressing N-terminally tagged GALC constructs. A) Examples of confocal images containing transfected and untransfected cells illustrating undetectable endogenous GALC staining. Cells were plated onto glass coverslips, fixed and immunostained using monoclonal antibody against GALC (green) and the ER marker calreticulin (red). B) Representative confocal image of untransfected cells with data collected at high laser intensity to illustrate non-specific staining only. C) Representative confocal image of HeLa cells transfected with an N-terminally tagged construct of GALC to illustrate equivalent co-localization with the lysosomal marker cathepsin D (red).
